# Avian Influenza Viruses, Inflammation, and CD8^+^ T Cell Immunity

**DOI:** 10.3389/fimmu.2016.00060

**Published:** 2016-03-01

**Authors:** Zhongfang Wang, Liyen Loh, Lukasz Kedzierski, Katherine Kedzierska

**Affiliations:** ^1^Department of Microbiology and Immunology, Peter Doherty Institute for Infection and Immunity, University of Melbourne, Melbourne, VIC, Australia

**Keywords:** avian influenza virus, CD8+ T cells, inflammation, influenza, human, influenza disease

## Abstract

Avian influenza viruses (AIVs) circulate naturally in wild aquatic birds, infect domestic poultry, and are capable of causing sporadic bird-to-human transmissions. AIVs capable of infecting humans include a highly pathogenic AIV H5N1, first detected in humans in 1997, and a low pathogenic AIV H7N9, reported in humans in 2013. Both H5N1 and H7N9 cause severe influenza disease in humans, manifested by acute respiratory distress syndrome, multi-organ failure, and high mortality rates of 60% and 35%, respectively. Ongoing circulation of H5N1 and H7N9 viruses in wild birds and poultry, and their ability to infect humans emphasizes their epidemic and pandemic potential and poses a public health threat. It is, thus, imperative to understand the host immune responses to the AIVs so we can control severe influenza disease caused by H5N1 or H7N9 and rationally design new immunotherapies and vaccines. This review summarizes our current knowledge on AIV epidemiology, disease symptoms, inflammatory processes underlying the AIV infection in humans, and recent studies on universal pre-existing CD8^+^ T cell immunity to AIVs. Immune responses driving the host recovery from AIV infection in patients hospitalized with severe influenza disease are also discussed.

## Avian Influenza Viruses and Their Epidemiology

Avian influenza viruses (AIVs, which belong to type A) circulate in birds as well as avian viral reassortants capable of infecting humans. Although AIV infections of wild birds are mainly asymptomatic, wild birds carry AIVs in their intestines and, thus, shed the virus via feces, saliva, and nasal secretions. The first avian infection was recorded in 1878 in Italy, named a “fowl plague” and subsequently isolated from chickens in 1902. Aquatic birds are considered a natural reservoir of AIVs (1) and, to date, all the AIV subtypes were isolated from aquatic birds, with the exception of H17N10 and H18N11 found in bats (2). Based on the pathogenicity in chickens, AIVs are classified into low pathogenic avian influenza viruses (LPAIVs) and highly pathogenic avian influenza viruses (HPAIVs). LPAIVs, commonly found in wild birds, cause only mild disease in domestic birds, while HPAIVs cause severe influenza symptoms, affect respiratory tract as well as internal organs and lead to a high mortality rate in poultry. So far, influenza H5 and H7 subtypes, originating from non-pathogenic precursor viruses, are the only HPAIVs (3). Primary outbreaks of HPAIV in poultry (turkey and chicken) have been reported 17 times between 1959 and 2000 and 8 times since 1990. They resulted in devastating losses of birds as millions of animals had to be culled to control those AIV outbreaks (4).

Out of all the AIVs, only H5N1, H7N2 (5), H7N3 (6, 7), H7N7 (8, 9), H9N2 (10), H7N9 (11), H5N6 (12), H6N1 (13), H10N7 (14, 15), and H10N8 (16) are known to cause human infections (Table 1). Generally, human infections with H7N2, H7N3, H7N7, and H6N1 result in mild clinical symptoms. However, some AIVs, including H5N1 and more recently, H7N9 strains, cause severe disease and high mortality rates in humans. This is mainly due to the absence of pre-existing AIV-specific antibodies (17–20), although both anti-H5 and H7-antibodies can be detected during the course of infection (21, 22). The highly pathogenic H5N1 virus attracted widespread attention in 1997, when a H5N1 outbreak in Hong Kong resulted in 18 human infections and 6 deaths. Subsequent outbreaks of the HPAIV H5N1 subtype occurred in China, Indonesia, Malaysia, Vietnam, and Thailand poultry industries in the early 2004, resulting in 13 human cases with 9 deaths in Vietnam, and 27 cases with 20 deaths in Thailand (23). Since then, multiple reassortant H5 HPAIV strains have spread across Asia and into Europe, the Middle East and Africa, giving rise to multiple subtypes (H5N2, H5N5, H5N6, and H5N8). Humans in close contact with AIV-infected poultry, and on rare occasion with other AIV-infected humans, can become infected. Globally, the total number of H5N1 cases reached 842 with 447 deaths. Thus, the fatality rate is >50% for H5N1 infections. Although, several studies addressed the calculations of more realistic rates of H5N1 infections, taking into account mild asymptomatic cases and low transmissibility, these are still estimated to be >30% (24). Countries that had the highest number of documented cases of H5N1 include Egypt, Indonesia, Vietnam, Cambodia, and China, where the total numbers ranged from 52 to 344 (WHO/GIP; data obtained on the 23 June 2015). The overall prevalence of H5 viruses increased in China since 2010 (25), with H5-type HPAIV, H5N6 and H5N8 being prevalent among China’s domestic waterfowl. Avian H5N6 influenza virus is a low virulent strain in poultry that has caused several outbreaks in chickens in China, Vietnam, and Laos during 2014 and 2015. Two cases of H5N6 were reported recently in humans in China and the clinical manifestations were severe (12, 26).

**Table 1:** **Summary of human infections caused by the avian influenza viruses**.

Influenza strain	Year/location	Cases/fatalities	Clinical manifestation	Reference
H5N1	1997/Hong Kong	18/6	Flu-like symptoms, pneumonia	(27)
	2003/Hong Kong	2/1	Pneumonia	(28)
	2003/China	1/1	Pneumonia	(29)
	2003–2015/Vietnam, Thailand, Cambodia, Indonesia, China, Azerbaijan, Djibouti, Egypt, Iraq, Nigeria, Turkey, Laos, Myanmar, Pakistan, Bangladesh, Canada	844/449	Flu-like symptoms, pneumonia	WHO/GIP, data in HQ as of 13 November 2015
H5N6	2014/China	2/1	Severe respiratory symptoms	(12, 26)
H6N1	2013/Taiwan	1/0	Flu-like symptoms	(13)
H7N2	2002/USA	1/0	Flu-like symptoms	(30)
	2003/USA	1/0	Respiratory tract infection	(30)
	2007/UK	1/0	Conjunctivitis	(31)
H7N3	2004/Canada	2/0	Conjunctivitis	(32)
	2006/UK	1/0	Conjunctivitis	(33)
	2012/Mexico	2/0	Conjunctivitis	(34)
H7N7	1980/USA	3/0	Conjunctivitis	(35)
	1995/UK	1/0	Conjunctivitis	(36)
	2003/Netherlands	89/1	Conjunctivitis, pneumonia, flu-like symptoms	(37)
H7N9	2013–2015/China	665/229	Range from mild to severe respiratory symptoms	WHO, CDC
H9N2	1998/China	5/0	Flu-like symptoms	(38)
	1999/Hong Kong	2/0	Flu-like symptoms	(39)
	2003/Hong Kong	1/0	Flu-like symptoms	(40)
	2007/Hong Kong	1/0	Flu-like symptoms	(30)
	2009/Hong Kong	2/0	Flu-like symptoms	(41)
	2011/Bangladesh	1/0	Flu-like symptoms	(42)
H10N7	2004/Egypt	2/0	Flu-like symptoms	(14)
	2010/Australia	2/0	Conjunctivitis, respiratory tract symptoms	(15)
H10N8	2013/China	3/2	Pneumonia	(16)

H7 subtype AIV has caused numerous outbreaks in poultry in Europe and the Americas. In 2003, H7N7 emerged in poultry in the Netherlands, and then spread to Belgium and Germany (43, 44), leading to the culling of over 30 million birds and resulting in >80 human cases, with one fatality (45). In 2013 in Italy, monitoring of the workers from a recent H7N7 outbreak at a poultry farm revealed three cases of H7N7 human mild infections. H7N3 viruses were reported in poultry in several countries, including Chile and Canada, and a recent 2013 outbreak of HPAIV H7N3 virus in Mexico led to the culling of >20 million birds and two confirmed human mild infections (6). Since March 2013, a novel reassortant A/H7N9 virus crossed host barrier from birds to humans, resulting in 657 human cases with 38% mortality (46). This is striking given that the 1918 Spanish influenza pandemic had a fatality rate of >2.5% and was considered to be extremely severe (47, 48). Although H7N9 virus causes severe human infections with a 98% hospitalization rate, H7N9 is a low pathogenic influenza virus in birds and can be found in live poultry markets across China, but rarely at the poultry farms.

In 2013, H10N8 virus emerged from live poultry markets. It originated from the internal genes of enzootic H9N2 viruses in chickens, and surface genes of H10 and N8 viruses from domestic ducks. It caused three severe infections in humans, leading to two deaths (16). Interestingly, internal genes of both H7N9 and H10N8 originated from H9N2 virus (49), an ongoing reassortant with local chicken H9N2 strains, thought to have a high epidemic or pandemic potential. However, it is not fully understood how these H9N2 chicken viruses facilitated the genesis of the newly emerged H7N9 virus (50). Since 1990s, H9N2 avian influenza viruses became well established in domestic poultry across Asia and Europe (51). Although of low pathogenicity, five human cases of H9N2 infection were reported during 1998 and 2008 [CIDRAP: Avian Influenza (Bird Flu): Implications for Human Disease]. H9N2 AIVs rapidly differentiated giving raise to more than 102 genotypic variants being recognized based on the nomenclature system (52). Notably, H9N2 viruses belonging to genotype 57 (G57) have a selective advantage in chickens, with increased infectivity and ability to escape selective pressure via antigenic shift. G57 AIVs caused the widespread H9N2 outbreaks among chickens during 2010–2013 in China (50). The virus became predominant in H9N2-vaccinated farm chickens, caused widespread outbreaks prior to the emergence of the H7N9 virus, increased reassortment events between H9N2 and other viral subtype, and finally provided all of the internal genes to the novel H7N9 virus. The prevalence and high mutation rate of the H9N2 influenza viruses at the poultry farms, their capacity to provide internal genes to other avian viruses, and adaptation to the human host could provide an important early warning of the emergence of novel reassortants with pandemic potential.

## Risk of Influenza Pandemics

Wide distribution of AIVs in poultry, occasional jumping across the host barrier to humans and the continuing evolution raise concerns about AIV’s pandemic potential. Nowadays, H9N2 and H5-type AIVs have nearly a global distribution in birds, and H7N9 and H10N8 viruses are widely circulating in live poultry markets in China (25). Occasional human-to-human transmission of H5, H6, H7, H9, and H10 AIV and their continuous reassortment with local chicken AIV strains (especially local H9N2 strains) have a potential of becoming influenza pandemic. H7N9, in particular, appears to be fulfilling the requirements for a potential influenza pandemic virus: (1) crosses the host barriers and is widespread in live poultry markets; (2) adapts to human host and further reassorts with local H9N2, while being a “low-pathogenicity” virus in chickens; (3) there are no pre-existing neutralizing antibodies (NAbs) at the population level. Overall, there is an urgent need to understand the pathogenesis and immunity of AIVs in humans, especially for those strains that can cross the host barrier to humans.

## Clinical Features of Severe Avian Influenza Disease

During seasonal influenza epidemics, elderly individuals (65+ years of age) showed higher rates of hospitalization (53). Severe disease from H5N1 infection was observed across different age groups, although higher hospitalization was found in several countries, including Vietnam and Indonesia in children and young adults (54). Conversely, during the first wave of H7N9 infections, more severe disease was observed in males aged >59 years old (55). These men shopped at the live bird markets in China and, therefore, were more likely exposed to H7N9 viruses. Furthermore, during aging both the innate and adaptive immune systems undergo immunosenesence, and results in decreased responsiveness to influenza vaccination and infection (56, 57). Some of the clinical and pathological features of severe A/H7N9 disease induced by AIVs infections include multiple organ failure, leukopenia, lymphopenia, viral dissemination in extra-pulmonary sites, and prolonged viral shedding of H7N9 viral RNA in feces and urine (11, 58–62). Indeed, high viral loads were also associated with death in H5N1-infected patients (63). During the recent H7N9 epidemic, decreased viral load was associated with recovery and outcome (59). Fatalities from avian influenza often result from pneumonia and acute respiratory syndrome (ARS) (64). Most hospitalized patients present with viral pneumonia, and the prevalence of bacterial co-infections infections is limited (51, 65). However, many hospitalized patients undergo broad-spectrum antibiotic treatment (20, 64), which possibly confound the detection of bacterial co-infections. This is in contrast to the 1918 pandemic during which secondary bacterial pneumonia was the leading cause of mortality (62, 66). Additionally, co-morbidities and underlying medical conditions, including type I/II diabetes and hypertension, were highly prevalent in the hospitalized cases of H7N9 influenza (22, 64). Interestingly, mild influenza disease was observed in young children with documented cases of H5N1 and H7N9 infections (67). Clinically, these children presented with fever, but no ARS-associated symptoms, such as pulmonary edema, were observed (67). However, the study had a small sample size (*n* = 2 ≤ 4), and the inflammatory milieu was not addressed. These observations warrant more analyses on the age-associated changes in the human immune system.

High fatality rates in AIV cases raise the question of how the avian viruses interact with the host immune system to cause such severe disease. Indeed, high viral titers and exuberant inflammatory responses contribute to fatal and severe avian influenza clinical disease. The mechanisms driving this in humans are likely to involve both intrinsic viral pathogenicity factors and dysregulation of inflammatory responses. The following sections of this review focus on the host immune responses to AIVs in humans.

## Inflammation during Avian Influenza Infection

Inflammation acts as a double-edged sword during viral infections. While inflammation can promote the recruitment of immune cells, uncontrolled and exacerbated inflammation, as observed in many AIV human cases, is associated with systemic edema and extensive tissue damage. It is well established that human infection with avian viruses, including H7 and H5 subtypes, can lead to “cytokine storms.” Analyses of serum and plasma from hospitalized patients revealed that hypercytokinemia of pro-inflammatory mediators (MCP-1, MIF, IL-6, IL-8, IP-10) and angiogenic/growth factors (HGF, SCF) is a common factor shared by H5N1 and H7N9 infections (63, 68, 69), and can be linked to fatal disease outcomes (68). Furthermore, analyses of bronchoalveolar lavage samples from hospitalized H7N9-infected patients showed elevated levels (by 100- to 1000-fold) of pro-inflammatory modulators, such as IL-6, IL-8, MIP-1β, or IL-1β (69). Thus, plasma/serum cytokine analyses give an indication of spillover into the periphery of patients with severe infection, although the concentrations are considerably lower. Additionally, airway epithelial cells infected with H5N1 viruses contribute to this inflammatory milieu (70, 71).

Similar findings were observed in macaques, ferrets, and mice challenged with HPAIV H5N1. Extensive tissue damage and hypercytokinemia are consistently present in H5N1-inoculated animals, although HPAIV-infected mice have greater inflammatory responses in the brain (72–75). Indeed, the clinical observations of hospitalized patients and animal studies to date showed that high levels of pro-inflammatory responses were strongly associated with severe disease from both H5N1 and H7N9 infections. Interestingly, anti-inflammatory molecules, including IL-10, were detected in serum of H5N1-infected patients (63). Furthermore, limited production of anti-viral type I IFN, which in part serves to limit viral replication, was observed in respiratory epithelial cells infected with H5 or H7 subtype *in vitro* (76, 77). This indicates that there may be an attempt by the immune system to modulate inflammation, although this could be too little too late. Murine studies demonstrated that blocking pro-inflammatory cytokines, such as TNF, IL-6, and CCL2, did not improve survival of mice challenged with HPAIV (78), suggesting that multiple mechanisms, in addition to inflammation, are likely to exacerbate pathology. Mechanistically, blocking pro-­inflammatory mediators in humans has not yet been preformed for AIVs. Further studies examining the use of immunomodulating cytokines and chemokines are warranted if alternative therapies are to be utilized for the treatment of severe influenza disease.

## The Innate Immune Responses during Avian Influenza Infection

The innate immune response, composed of both cellular and soluble mediators, forms the first line of defense during viral infections. Indeed, the protective role of innate immunity is clearly demonstrated during HPAI infection of birds whereby, ducks exhibit asymptomatic or mild disease, while chickens become ill. Following H5N1 infection, primary lung cells isolated from chickens showed highly elevated immune and pro-inflammatory responses compared to duck cells, which appeared to be mediated via inhibition of STAT-3 (79). Another study also found that RIG-I is absent in chickens, providing a plausible explanation for their increased susceptibility to influenza viruses as compared with ducks (80).

In humans, immunohistological analyses of lung tissues in five fatal cases of H5N1 showed an influx of neutrophils into the alveolar spaces, while TNF and IL-6 were detected in monocytes/macrophages *in situ* (81). The role of monocyte-derived macrophages (MDMs) and alveolar macrophages in protection versus pathology in avian influenza remains controversial. Robust pro-inflammatory responses, including TNF and IP-10 were detected in culture supernatants in H5N1-infected human MDMs *in vitro* (82–84). This was dependent on the viral isolate and independent of viral replicative capacity in MDMs (82–84). Conversely, alveolar macrophages isolated from human lungs showed limited cytokine secretion after HPAIV infection (85). The latter study demonstrated that the permissiveness to infection was lower in alveolar macrophages than in MDMs (85). These data indicate differential roles of tissue-resident macrophages versus circulating and differentiated monocytes during avian influenza infections. Although, H7N9 viruses isolated from humans can replicate more efficiently in cultured primary alveolar macrophages, they induce lower levels of cytokine production, including TNF and CCL5 as compared to H5N1 viruses (86).

Indeed, during the steady state, tissue macrophages have been shown to provide homeostatic functions, including resolution of inflammation via removal of apoptotic debris and cells (87). Conversely, MDMs have an inflammatory effector profile, suggesting that they have the potential to contribute to inflammation-induced pathology (87, 88). Together, these studies highlight the importance of understanding how microenvironments drive differential levels of inflammation during avian influenza infection.

Natural killer (NK) cells, which comprise up to 10% of the lymphocyte compartment, contribute to immune homeostasis and viral immunity in both cancer and chronic viral infections. Their activation is regulated by the interaction of combinations of activating and inhibitory receptors with self and altered-self ligands, e.g., killer Ig-like receptors, CD16 (Fcγ receptor), NKp46 (89–91). Expanded numbers of NK cells were observed in H7N9-infected hospitalized patients with prolonged hospital stays. Furthermore, in these patients robust IFNγ responses were observed following *ex vivo* H7N9 virus stimulations, indicating preserved effector functionality (22). Additionally, antibody-dependent cell cytotoxic (ADCC) antibody isotypes could be detected in H7N9- and H5N1-infected patients (92). Recent studies have also shown that NK cells from healthy influenza-seropositive donors, can be activated via ADCC, where NK cells robustly upregulate IFNγ production and the surrogate marker of degranulation, CD107a (89). In the absence of neutralizing H5-antibodies, these responses were also cross-reactive with H5N1 hemagglutinin (84). Together, these studies demonstrate a role of NK cells during avian influenza virus infection and their potential to kill AIV-infected cells and aid in the viral control.

Other innate cellular components include the innate-like T cells, γδ T cell, NKT, mucosal-associated invariant T cells, which express re-arranged T cell receptors, and also the innate-lymphoid cells, which lack re-arranged receptors. Collectively, these innate-like cells produce inflammatory and immune-modulating cytokines (e.g., IFNγ, TNF, IL-22, IL-17, IL-33), which are found in the periphery and can be enriched in peripheral tissues (93–96). Although there is a paucity of data regarding their role in AIV infection, the immunoregulatory roles of ILCs and NKTs were demonstrated in murine models of influenza. Both ILCs and NKTs have been shown to aid in the protection of the lung epithelium during IAV infection via the production of IL-33 and IL-22, respectively (97, 98). An example of cytolytic potential during avian influenza virus infection comes from human Vγ9δ2 T cells, where the killing of H5N1-infected MDMs were demonstrated when expanded prior with a TCR-cognate phosphoantigen (99). Together, innate lymphocytes may be utilized as alternative biological therapies for severe avian influenza.

## CD8^+^ T Cell Immunity Against Avian Influenza Viruses

Influenza virus-specific CD8^+^ cytotoxic T lymphocytes (CTLs) provide protection against severe influenza disease, accelerate viral clearance, and promote rapid host recovery (22, 100–102). CTL-based heterosubtypic immunity against seasonal influenza viruses has been extensively studied in animal models (103, 104). In murine models, primary influenza-specific CTLs emerge around day 7, peak around day 10, and then contract via apoptosis. This is in response to the influenza virus replication, which peaks at days 3–5 and gets resolved around days 8–10 (105–107). A few studies were performed to investigate how CD8^+^ T cell responses are induced during HPAIV (e.g., H5N1) infection in mice and whether virus-specific CD8^+^ T cells play a protective or immunopathologic role during a primary HPAIV infection. Hatta et al. showed that different virulent H5N1 viruses had different replication kinetics in mice and that rapid replication could outpace the CD8^+^ T cell response (108). Some studies reported that H7N9 replication in mice peaked at day 5 (109) and cleared around day 8 (110), while others reported that during the primary infection of B6 mice, virus peaked on day 6, decreased by days 8–10, and cleared by day 12 (111). In any case, this appears to differ from human H7N9 disease, where H7N9 viral RNA could still be detected in nasal swabs, blood, urine, or feces at days 8–14 following disease onset. Since the virus replication kinetics and the pathogenicity of HPAIV in mice differ to those found in humans, it is of key importance to understand the influenza viral replication and CTL responses in humans.

Published reports provide evidence that prominent pre-­existing influenza-specific CD8^+^ or CD4^+^ memory T cell pools are associated with more rapid recovery from experimentally mild H3N2 or naturally occurring H1N1-2009 influenza virus infection, as manifested by milder disease symptoms, shorter illness time, diminished viral shedding, and less transmission (100, 101, 112, 113). However, the exact kinetics and the role of CD8^+^ or CD4^+^ T cell responses in the recovery from infection, especially severe AIV infection, is not well understood. Recently, Sridhar et al. showed that the pre-existing IFN-γ-producing memory CD8^+^ T cells directed at the conserved epitopes inversely correlated with disease illness and symptom scores (101). Wilkinson et al. reported that influenza-specific T cell response increased largely at day 7 when virus was completely undetectable in nasal samples, while serum antibodies were present (112). Pre-existing memory CD4^+^ T cells, capable of killing virus-infected cells responded to influenza internal proteins and inversely correlated with less virus shedding and lower disease scores. The above-mentioned studies used the household cohort approach during the 2009/2010 pH1N1 infection season (101) and the human influenza challenge with experimentally low dose of seasonal H3N2 and H1N1 infections (112), with viral clearance around days 6–8 after influenza infection (114). This might, however, differ to the viral replication and the resultant cellular immune responses following natural severe AIV infection.

The kinetics of CD8^+^ T cell responses in human influenza infection has been addressed by Rimmelzwaan’s laboratory. Hillaire et al. reported that virus-specific CD8^+^ T cells peaked within 1 week after influenza virus infection and then contracted rapidly (115), consistent with the observations in mice (116). However, Ennis et al. found that virus-specific CD8^+^ T cells expanded around days 6–14 post-infection, and then declined around day 21 after infection (117), whereas Wagar et al. showed that total IFNγ^+^CD8^+^ T cells peak at around 3 weeks after the disease onset in case of pH1N1 infection. Interestingly, the CD8^+^ T cell number moderately decreased around day 78 and reached the baseline level at day 700 post-disease onset (118), consistent with Ennis’ report. As mentioned previously, the new AIV H7N9 could replicate actively between days 8 and 14 (59), which differs greatly from the mild infection in humans reported for pandemic H1N1 (101) and experimental H3N2 infection (112). This raises the following questions: (1) weather the kinetics of CD8^+^/CD4^+^ responses during AIV infection are similar to those observed during a mild influenza disease; (2) whether CD8^+^ T cell responses are mainly protective or could also be immunopathogenic; (3) whether the expanded CD8^+^ T cell responses are derived predominantly from the memory pools or possibly recruited from naïve precursors; and (4) what is the relationship between CD8^+^ T cells, CD4^+^ T cells, and humoral immunity.

The reports that looked at human CD8^+^ T cell responses elicited to HPAIV are scarce and focused mainly on pre-existing and cross-protective immunity (Table 2). Early reports indicated that memory CD8^+^ (and CD4^+^) T cells from healthy individuals with no documented history of prior exposure to avian influenza could recognize epitopes from avian-derived viruses, including 1997 Hong Kong H5N1 (119). The cross-reactive nature of influenza-specific CD8^+^ T cells was further demonstrated by several groups (120–122), which reported heterosubtypic memory T cell responses against avian H5N1 in healthy individuals without prior exposure to AIV. This heterosubtypic immunity was proposed to provide at least some level of protection toward the new emerging, possibly pandemic, influenza AIV strains. Nevertheless, there are no reports addressing the role of CD8^+^ T cells in individuals infected with primary H5N1 virus in humans. Similarly, it has been reported that a high percentage of CD8^+^ T cells specific for seasonal (H1N1 and H3N2) and pandemic (H1N1) viruses cross-react with the newly emerged H7N9 AIV and suggested that those cross-reactive CD8^+^ T cells can reduce disease severity and virus spread within the population (123). However, the population coverage of these broadly cross-reactive pre-existing CTLs varied greatly across various ethnicities, indicating that especially indigenous populations are at the higher risk of succumbing to H7N9 infection (124). Our recent study showed the importance of CD8^+^ T cell responses against H7N9 AIV in the absence of pre-existing NAbs (22). We showed that H7N9-infected patients discharged from the Shanghai Public Health Clinical Centre within 2–3 weeks following admission had early prominent H7N9-specific CD8^+^ T cell responses. On the other hand, individuals with prolonged hospitalization showed late recruitment of CD8^+^/CD4^+^ T cells and antibodies simultaneously (recovery by week 4), augmented by prominent NK cell responses (recovery >30 days). By contrast, those with fatal disease outcomes had minimal influenza-specific immunity and little evidence of T cell activation at the transcriptional level (69).

**Table 2:** **Relevant publications assessing human T cell immunity toward avian H5N1 and H7N9 influenza viruses**.

Reference	Avian influenza subtype	T cells	Responses toward avian viruses
Jameson et al. (119)	H5N1	CTLs reactive to H1N1, H2N2, and H3N2, obtained from healthy individuals from USA	Cross-reactivity with H5N1
Lee et al. (122)	H5N1	CTLs reactive to H3N2 obtained from healthy individuals from Vietnam and UK	Cross-reactivity with H5N1
Goy et al. (120)	H5N1	CTLs reactive to H1N1 and H3N2 obtained from healthy individuals from Australia	Cross-reactivity with H5N1
Kreijtz et al. (121)	H5N1	*In vitro* expanded CTLs toward H3N2 obtained from healthy HLA-typed donors from the Netherlands	Cross-reactivity with H5N1-infected BLCL
van de Sandt et al. (123)	H7N9	CTLs expanded *in vitro* toward H1N1 (seasonal and pandemic) or H3N2 (seasonal) from healthy HLA-typed donors from the Netherlands	CTL cross-reactivity with H7N9-peptide loaded BLCLs
Quinones-Parra et al. (124)	H7N9	*In vitro* peptide-expanded PBMCs obtained from healthy HLA-typed individuals from Australia	CTL cross-reactivity with H7N9-derived immunogenic peptides is restricted by certain HLA haplotypes and varies across ethnicities
Chen et al. (20)	H7N9	PBMCs obtained from H7N9-infected patients in China	High numbers of peripheral blood T lymphocytes (CD4^+^ and CD8^+^) correlate with better clinical outcomes
Wang et al. (22)	H7N9	*Ex vivo* longitudinal analyses of PBMCs obtained from hospitalized H7N9-infected patients in Shanghai, China	Rapid recovery from H7N9 infection is associated with early CD8^+^ T cell responses

However, all of the above human studies are based on the assessment of PBMCs within the peripheral blood, which might not totally reflect what happens at the site of infection. Although the previous reports from mice studies (125, 126) showed that blood CD8^+^ T cell response could be a surrogate for the CD8^+^ T cell response in the lung and lymph nodes, there are still no published studies in humans that investigate whether similar kinetics occur within the blood and lungs during human influenza infection. In addition, it is difficult to know how many lung resident memory T cells are present following the natural influenza virus infection and how protective they are against the heterologous challenge in humans.

## Conclusion

Taken together, it seems that the pathogenicity and virulence of AIVs in humans are determined by the interactions between the virus and the host immune response. For mild infections, whether caused by seasonal influenza viruses or occasional asymptomatic AIVs, the pre-existing CD8^+^ and/or CD4^+^ T cells can provide a great level of protection (101, 112, 115). In this situation, innate immunity provides a rapid first-line immune defense, slows down the virus, and assists development of adaptive immunity, without any damaging “cytokine storm” effect. This is then followed by adaptive immunity capable of clearing the viral infection and promoting host recovery. However, in case of a severe human influenza infection, caused by either seasonal influenza viruses or HPAIV, due to as yet unknown reasons, infected people are more susceptible to severe outcome. High replication of AIV may either inhibit the innate immunity or drive the overactive innate immune response. This may then interfere with generation of rapid and effective adaptive immunity and, thus, subsequently result in detrimental cytokine storm. Defective innate and/or adaptive immune mechanisms cannot control the infection in a timely fashion, as shown in our H7N9 study (22). Delayed CD8^+^ T cell responses in patients with prolonged hospital stays could have also resulted from the lack of robust cross-strain protective memory pools, thereby reflected the need to recruit the naïve CD8^+^ T cell precursors into the primary influenza response rather than rapidly recalling the available memory CD8^+^ T cells. Thus, establishment of robust memory pools appears to be of a great importance for the rapid recruitment of adaptive immune responses after HPAIV, so that the innate immunity (and inflammation) does not get to the point that is immunosuppressive for cellular immunity and, thus, cannot control the rapidly replicating virus (22).

## Author Contributions

ZW, LL, LK and KK wrote and edited the manuscript.

## Conflict of Interest Statement

The authors declare that the research was conducted in the absence of any commercial or financial relationships that could be construed as a potential conflict of interest.

## References

[1] Peiris JS de Jong MD Guan Y. Avian influenza virus (H5N1): a threat to human health. Clin Microbiol Rev (2007) 20(2):243–67. 10.1128/CMR.00037-06 17428885 PMC1865597

[2] Shi Y Wu Y Zhang W Qi J Gao GF. Enabling the ‘host jump’: structural determinants of receptor-binding specificity in influenza A viruses. Nat Rev Microbiol (2014) 12(12):822–31. 10.1038/nrmicro3362 25383601

[3] Alexander DJ. An overview of the epidemiology of avian influenza. Vaccine (2007) 25(30):5637–44. 10.1016/j.vaccine.2006.10.051 17126960

[4] Alexander DJ. A review of avian influenza in different bird species. Vet Microbiol (2000) 74(1–2):3–13. 10.1016/S0378-1135(00)00160-7 10799774

[5] Ostrowsky B Huang A Terry W Anton D Brunagel B Traynor L Low pathogenic avian influenza A (H7N2) virus infection in immunocompromised adult, New York, USA, 2003. Emerg Infect Dis (2012) 18(7):1128–31. 10.3201/eid1807.111913 22710273 PMC3376803

[6] Centers for Disease Control and Prevention. Notes from the field: highly pathogenic avian influenza A (H7N3) virus infection in two poultry workers – Jalisco, Mexico, July 2012. MMWR Morb Mortal Wkly Rep (2012) 61(36):726–7. 22971746

[7] Skowronski DM Tweed SA Petric M Booth T Li Y Tam T Human illness and isolation of low-pathogenicity avian influenza virus of the H7N3 subtype in British Columbia, Canada. J Infect Dis (2006) 193(6):899–900;authorrely900. 10.1086/500219 16479527

[8] Fouchier RA Schneeberger PM Rozendaal FW Broekman JM Kemink SA Munster V Avian influenza A virus (H7N7) associated with human conjunctivitis and a fatal case of acute respiratory distress syndrome. Proc Natl Acad Sci U S A (2004) 101(5):1356–61. 10.1073/pnas.0308352100 14745020 PMC337057

[9] Puzelli S Rossini G Facchini M Vaccari G Di Trani L Di Martino A Human infection with highly pathogenic A(H7N7) avian influenza virus, Italy, 2013. Emerg Infect Dis (2014) 20(10):1745–9. 10.3201/eid2010.140512 25271444 PMC4193179

[10] Capua I Alexander DJ Avian influenza and human health. Acta Trop (2002) 83(1):1–6. 10.1016/S0001-706X(02)00050-5 12062786

[11] Gao R Cao B Hu Y Feng Z Wang D Hu W Human infection with a novel avian-origin influenza A (H7N9) virus. N Engl J Med (2013) 368(20):1888–97. 10.1056/NEJMoa1304459 23577628

[12] Yang ZF Mok CK Peiris JS Zhong NS Human infection with a novel avian influenza A(H5N6) virus. N Engl J Med (2015) 373(5):487–9. 10.1056/NEJMc1502983 26222578

[13] Wei SH Yang JR Wu HS Chang MC Lin JS Lin CY Human infection with avian influenza A H6N1 virus: an epidemiological analysis. Lancet Respir Med (2013) 1(10):771–8. 10.1016/S2213-2600(13)70221-2 24461756 PMC7164810

[14] Avian Influenza Virus A(H10N7) Circulating Among Humans in Egypt. Pan American Health Organization PAHO (2004). Available from: http://new.paho.org/hq/dmdocuments/2010/Avian_Influenza_Egypt_070503.pdf

[15] Arzey GG Kirkland PD Arzey KE Frost M Maywood P Conaty S Influenza virus A (H10N7) in chickens and poultry abattoir workers, Australia. Emerg Infect Dis (2012) 18(5):814–6. 10.3201/eid1805.111852 22516302 PMC3358073

[16] Liu M Li X Yuan H Zhou J Wu J Bo H Genetic diversity of avian influenza A (H10N8) virus in live poultry markets and its association with human infections in China. Sci Rep (2015) 5:7632. 10.1038/srep07632 25591167 PMC5379002

[17] Tran TH Nguyen TL Nguyen TD Luong TS Pham PM Nguyen V Avian influenza A (H5N1) in 10 patients in Vietnam. N Engl J Med (2004) 350(12):1179–88. 10.1056/NEJMoa040419 14985470

[18] de Jong MD Bach VC Phan TQ Vo MH Tran TT Nguyen BH Fatal avian influenza A (H5N1) in a child presenting with diarrhea followed by coma. N Engl J Med (2005) 352(7):686–91. 10.1056/NEJMoa044307 15716562

[19] Hien TT de Jong M Farrar J Avian influenza – a challenge to global health care structures. N Engl J Med (2004) 351(23):2363–5. 10.1056/NEJMp048267 15575048

[20] Chen Y Liang W Yang S Wu N Gao H Sheng J Human infections with the emerging avian influenza A H7N9 virus from wet market poultry: clinical analysis and characterisation of viral genome. Lancet (2013) 381(9881):1916–25. 10.1016/S0140-6736(13)60903-4 23623390 PMC7134567

[21] Rowe T Abernathy RA Hu-Primmer J Thompson WW Lu X Lim W Detection of antibody to avian influenza A (H5N1) virus in human serum by using a combination of serologic assays. J Clin Microbiol (1999) 37(4):937–43. 10074505 10.1128/jcm.37.4.937-943.1999 PMC88628

[22] Wang Z Wan Y Qiu C Quinones-Parra S Zhu Z Loh L Recovery from severe H7N9 disease is associated with diverse response mechanisms dominated by CD8(+) T cells. Nat Commun (2015) 6:6833 10.1038/ncomms7833 25967273 PMC4479016

[23] Ungchusak K Auewarakul P Dowell SF Kitphati R Auwanit W Puthavathana P Probable person-to-person transmission of avian influenza A (H5N1). N Engl J Med (2005) 352(4):333–40. 10.1056/NEJMoa044021 15668219

[24] Li FC Choi BC Sly T Pak AW. Finding the real case-fatality rate of H5N1 avian influenza. J Epidemiol Community Health (2008) 62(6):555–9. 10.1136/jech.2007.064030 18477756

[25] Su S Bi Y Wong G Gray GC Gao GF Li S. Epidemiology, evolution, and recent outbreaks of avian influenza virus in China. J Virol (2015) 89(17):8671–6. 10.1128/JVI.01034-15 26063419 PMC4524075

[26] Pan M Gao R Lv Q Huang S Zhou Z Yang L Human infection with a novel highly pathogenic avian influenza A (H5N6) virus: virological and clinical findings. J Infect (2015) 72(1):52–9. 10.1016/j.jinf.2015.06.009 26143617

[27] Centers for Disease Control and Prevention. Isolation of avian influenza A(H5N1) viruses from humans – Hong Kong, May-December 1997. MMWR Morb Mortal Wkly Rep (1997) 46(50):1204–7. 9414153

[28] Peiris JS Yu WC Leung CW Cheung CY Ng WF Nicholls JM Re-emergence of fatal human influenza A subtype H5N1 disease. Lancet (2004) 363(9409):617–9. 10.1016/S0140-6736(04)15595-5 14987888 PMC7112424

[29] Zhu QY Qin ED Wang W Yu J Liu BH Hu Y Fatal infection with influenza A (H5N1) virus in China. N Engl J Med (2006) 354(25):2731–2. 10.1056/NEJMc066058 16790715

[30] Peiris JS Avian influenza viruses in humans. Rev Sci Tech Off Int Epiz (2009) 28(1):161–74. 10.20506/rst.28.1.1871 19618624

[31] E. Team. Avian influenza A/(H7N2) outbreak in the United Kingdom. Euro Surveill (2007) 12(22):3206. 17868584

[32] Tweed SA Skowronski DM David ST Larder A Petric M Lees W Human illness from avian influenza H7N3, British Columbia. Emerg Infect Dis (2004) 10(12):2196–9. 10.3201/eid1012.040961 15663860 PMC3323407

[33] Nguyen-Van-Tam JS Nair P Acheson P Baker A Barker M Bracebridge S Outbreak of low pathogenicity H7N3 avian influenza in UK, including associated case of human conjunctivitis. Euro Surveill (2006) 11(5):E0605042. 10.2807/esw.11.18.02952-en 16816456

[34] Notes from the Field. Highly pathogenic avian influenza A (H7N3) virus infection in two poultry workers – Jalisco, Mexico, July 2012. MMWR Morb Mortal Wkly Rep (2012) 61(36):726–7. 22971746

[35] Webster RG Geraci J Petursson G Skirnisson K Conjunctivitis in human beings caused by influenza A virus of seals. N Engl J Med (1981) 304(15):911 10.1056/NEJM198104093041515 7207529

[36] Kurtz J Manvell RJ Banks J Avian influenza virus isolated from a woman with conjunctivitis. Lancet (1996) 348(9031):901–2. 10.1016/S0140-6736(05)64783-6 8826845

[37] Koopmans M Wilbrink B Conyn M Natrop G van der Nat H Vennema H Transmission of H7N7 avian influenza A virus to human beings during a large outbreak in commercial poultry farms in the Netherlands. Lancet (2004) 363(9409):587–93. 10.1016/S0140-6736(04)15589-X 14987882

[38] Guo Y Li J Cheng X. [Discovery of men infected by avian influenza A (H9N2) virus]. Zhonghua Shi Yan He Lin Chuang Bing Du Xue Za Zhi (1999) 13(2):105–8. 12569771

[39] Peiris M Yuen KY Leung CW Chan KH Ip PL Lai RW Human infection with influenza H9N2. Lancet (1999) 354(9182):916–7. 10.1016/S0140-6736(99)03311-5 10489954

[40] Butt KM Smith GJ Chen H Zhang LJ Leung YH Xu KM Human infection with an avian H9N2 influenza A virus in Hong Kong in 2003. J Clin Microbiol (2005) 43(11):5760–7. 10.1128/JCM.43.11.5760-5767.2005 16272514 PMC1287799

[41] Cheng VC Chan JF Wen X Wu WL Que TL Chen H Infection of immunocompromised patients by avian H9N2 influenza A virus. J Infect (2011) 62(5):394–9. 10.1016/j.jinf.2011.02.007 21356238

[42] International Centre for Diarrhoeal Disease Research, Bangladesh/Government of The People’s Republic of Bangladesh, Outbreak of mild respiratory disease caused by H5N1 and H9N2 infections among young children in Dhaka, Bangladesh, 2011. HSB (2011) 9(2):5–12.

[43] Barclay WS Zambon M Pandemic risks from bird flu. BMJ (2004) 328(7434):238–9. 10.1136/bmj.328.7434.238 14751872 PMC324441

[44] Monto AS The threat of an avian influenza pandemic. N Engl J Med (2005) 352(4):323–5. 10.1056/NEJMp048343 15668220

[45] Van Reeth K. Avian and swine influenza viruses: our current understanding of the zoonotic risk. Vet Res (2007) 38(2):243–60. 10.1051/vetres:2006062 17257572

[46] Li Q Zhou L Zhou M Chen Z Li F Wu H Epidemiology of human infections with avian influenza A(H7N9) virus in China. N Engl J Med (2014) 370(6):520–32. 10.1056/NEJMoa1304617 23614499 PMC6652192

[47] Taubenberger JK Morens DM Influenza revisited. Emerg Infect Dis (2006) 12(1):1–2. 10.3201/eid1201.051442 16610160 PMC3291416

[48] Taubenberger JK Morens DM. 1918 Influenza: the mother of all pandemics. Emerg Infect Dis (2006) 12(1):15–22. 10.3201/eid1209.05-0979 16494711 PMC3291398

[49] Ma C Lam TT Chai Y Wang J Fan X Hong W Emergence and evolution of H10 subtype influenza viruses in poultry in China. J Virol (2015) 89(7):3534–41. 10.1128/JVI.03167-14 25589662 PMC4403437

[50] Pu J Wang S Yin Y Zhang G Carter RA Wang J Evolution of the H9N2 influenza genotype that facilitated the genesis of the novel H7N9 virus. Proc Natl Acad Sci U S A (2015) 112(2):548–53. 10.1073/pnas.1422456112 25548189 PMC4299237

[51] Yu L Wang Z Chen Y Ding W Jia H Chan JF Clinical, virological, and histopathological manifestations of fatal human infections by avian influenza A(H7N9) virus. Clin Infect Dis (2013) 57(10):1449–57. 10.1093/cid/cit541 23943822

[52] Bi Y Lu L Li J Yin Y Zhang Y Gao H Novel genetic reassortants in H9N2 influenza A viruses and their diverse pathogenicity to mice. Virol J (2011) 8:505. 10.1186/1743-422X-8-505 22050764 PMC3236014

[53] Fiore AE Uyeki TM Broder K Finelli L Euler GL Singleton JA Prevention and control of influenza with vaccines: recommendations of the Advisory Committee on Immunization Practices (ACIP). MMWR Morb Mortal Wkly Rep (2010) 59:1–62. 20689501

[54] WER Wkly Epidemiol Rec (2008) 83(46):413–20.19009715

[55] Arima Y Vong S World Health Organization Outbreak Response Team Human infections with avian influenza A(H7N9) virus in China: preliminary assessments of the age and sex distribution. Western Pac Surveill Response J (2013) 4(2):1–3. 10.5365/wpsar.2013.4.2.005 24015363 PMC3762971

[56] Goronzy JJ Weyand CM. Understanding immunosenescence to improve responses to vaccines. Nat Immunol (2013) 14(5):428–36. 10.1038/ni.2588 23598398 PMC4183346

[57] Shaw AC Goldstein DR Montgomery RR. Age-dependent dysregulation of innate immunity. Nat Rev Immunol (2013) 13(12):875–87. 10.1038/nri3547 24157572 PMC4096436

[58] Zhu Z Liu Y Xu L Guan W Zhang X Qi T Extra-pulmonary viral shedding in H7N9 Avian Influenza patients. J Clin Virol (2015) 69:30–2. 10.1016/j.jcv.2015.05.013 26209373

[59] Hu Y Lu S Song Z Wang W Hao P Li J Association between adverse clinical outcome in human disease caused by novel influenza A H7N9 virus and sustained viral shedding and emergence of antiviral resistance. Lancet (2013) 381(9885):2273–9. 10.1016/S0140-6736(13)61125-3 23726392

[60] Uiprasertkul M Kitphati R Puthavathana P Kriwong R Kongchanagul A Ungchusak K Apoptosis and pathogenesis of avian influenza A (H5N1) virus in humans. Emerg Infect Dis (2007) 13(5):708–12. 10.3201/eid1305.060572 17553248 PMC2738443

[61] Zhang Z Zhang J Huang K Li KS Yuen KY Guan Y Systemic infection of avian influenza A virus H5N1 subtype in humans. Hum Pathol (2009) 40(5):735–9. 10.1016/j.humpath.2008.08.015 19121843 PMC7112124

[62] Writing Committee of the Second World Health Organization Consultation on Clinical Aspects of Human Infection with Avian Influenza A (H5N1) Virus, Abdel-Ghafar AN Chotpitayasunondh T Gao Z Hayden FG Nguyen DH Update on avian influenza A (H5N1) virus infection in humans. N Engl J Med (2008) 358(3):261–73. 10.1056/NEJMra0707279 18199865

[63] de Jong MD Simmons CP Thanh TT Hien VM Smith GJ Chau TN Fatal outcome of human influenza A (H5N1) is associated with high viral load and hypercytokinemia. Nat Med (2006) 12(10):1203–7. 10.1038/nm1477 16964257 PMC4333202

[64] Gao HN Lu HZ Cao B Du B Shang H Gan JH Clinical findings in 111 cases of influenza A (H7N9) virus infection. N Engl J Med (2013) 368(24):2277–85. 10.1056/NEJMoa1305584 23697469

[65] Beigel JH Farrar J Han AM Hayden FG Hyer R de Jong MD of the World Health Organization consultation on human influenza: avian influenza A (H5N1) infection in humans. N Engl J Med (2005) 353(13):1374–85. 10.1056/NEJMra052211 16192482

[66] Brundage JF Shanks GD. Deaths from bacterial pneumonia during 1918-19 influenza pandemic. Emerg Infect Dis (2008) 14(8):1193–9. 10.3201/eid1408.071313 18680641 PMC2600384

[67] Yu X Zhang X He Y Wu H Gao X Pan Q Mild infection of a novel H7N9 avian influenza virus in children in Shanghai. Emerg Microbes Infect (2013) 2(7):e41 10.1038/emi.2013.41 26038475 PMC3820982

[68] Guo J Huang F Liu J Chen Y Wang W Cao B The serum profile of hypercytokinemia factors identified in H7N9-infected patients can predict fatal outcomes. Sci Rep (2015) 5:10942. 10.1038/srep10942 26028236 PMC4450576

[69] Wang Z Zhang A Wan Y Liu X Qiu C Xi X Early hypercytokinemia is associated with interferon-induced transmembrane protein-3 dysfunction and predictive of fatal H7N9 infection. Proc Natl Acad Sci U S A (2014) 111(2):769–74. 10.1073/pnas.1321748111 24367104 PMC3896201

[70] Chan MC Cheung CY Chui WH Tsao SW Nicholls JM Chan YO Proinflammatory cytokine responses induced by influenza A (H5N1) viruses in primary human alveolar and bronchial epithelial cells. Respir Res (2005) 6:135. 10.1186/1465-9921-6-135 16283933 PMC1318487

[71] Zeng H Belser JA Goldsmith CS Gustin KM Veguilla V Katz JM A(H7N9) virus results in early induction of proinflammatory cytokine responses in both human lung epithelial and endothelial cells and shows increased human adaptation compared with avian H5N1 virus. J Virol (2015) 89(8):4655–67. 10.1128/JVI.03095-14 25673714 PMC4442366

[72] Baskin CR Bielefeldt-Ohmann H Tumpey TM Sabourin PJ Long JP Garcia-Sastre A Early and sustained innate immune response defines pathology and death in nonhuman primates infected by highly pathogenic influenza virus. Proc Natl Acad Sci U S A (2009) 106(9):3455–60. 10.1073/pnas.0813234106 19218453 PMC2642661

[73] Govorkova EA Rehg JE Krauss S Yen HL Guan Y Peiris M Lethality to ferrets of H5N1 influenza viruses isolated from humans and poultry in 2004. J Virol (2005) 79(4):2191–8. 10.1128/JVI.79.4.2191-2198.2005 15681421 PMC546577

[74] Tumpey TM Lu X Morken T Zaki SR Katz JM. Depletion of lymphocytes and diminished cytokine production in mice infected with a highly virulent influenza A (H5N1) virus isolated from humans. J Virol (2000) 74(13):6105–16. 10.1128/JVI.74.13.6105-6116.2000 10846094 PMC112109

[75] Zitzow LA Rowe T Morken T Shieh WJ Zaki S Katz JM. Pathogenesis of avian influenza A (H5N1) viruses in ferrets. J Virol (2002) 76(9):4420–9. 10.1128/JVI.76.9.4420-4429.2002 11932409 PMC155091

[76] Belser JA Zeng H Katz JM Tumpey TM. Infection with highly pathogenic H7 influenza viruses results in an attenuated proinflammatory cytokine and chemokine response early after infection. J Infect Dis (2011) 203(1):40–8. 10.1093/infdis/jiq018 21148495 PMC3086437

[77] Zeng H Goldsmith C Thawatsupha P Chittaganpitch M Waicharoen S Zaki S Highly pathogenic avian influenza H5N1 viruses elicit an attenuated type I interferon response in polarized human bronchial epithelial cells. J Virol (2007) 81(22):12439–49. 10.1128/JVI.01134-07 17855549 PMC2169033

[78] Salomon R Hoffmann E Webster RG. Inhibition of the cytokine response does not protect against lethal H5N1 influenza infection. Proc Natl Acad Sci U S A (2007) 104(30):12479–81. 10.1073/pnas.0705289104 17640882 PMC1924467

[79] Kuchipudi SV Tellabati M Sebastian S Londt BZ Jansen C Vervelde L Highly pathogenic avian influenza virus infection in chickens but not ducks is associated with elevated host immune and pro-inflammatory responses. Vet Res (2014) 45:118 10.1186/s13567-014-0118-3 25431115 PMC4246556

[80] Barber MR Aldridge JR Jr Webster RG Magor KE. Association of RIG-I with innate immunity of ducks to influenza. Proc Natl Acad Sci U S A (2010) 107(13):5913–8. 10.1073/pnas.1001755107 20308570 PMC2851864

[81] Nakajima N Van Tin N Sato Y Thach HN Katano H Diep PH Pathological study of archival lung tissues from five fatal cases of avian H5N1 influenza in Vietnam. Mod Pathol (2013) 26(3):357–69. 10.1038/modpathol.2012.193 23174938 PMC9813393

[82] Cheung CY Poon LL Lau AS Luk W Lau YL Shortridge KF Induction of proinflammatory cytokines in human macrophages by influenza A (H5N1) viruses: a mechanism for the unusual severity of human disease? Lancet (2002) 360(9348):1831–7. 10.1016/S0140-6736(02)11772-7 12480361

[83] Sakabe S Iwatsuki-Horimoto K Takano R Nidom CA Le M Nagamura-Inoue T Cytokine production by primary human macrophages infected with highly pathogenic H5N1 or pandemic H1N1 2009 influenza viruses. J Gen Virol (2011) 92(Pt 6):1428–34. 10.1099/vir.0.030346-0 21367984 PMC3168279

[84] Friesenhagen J Boergeling Y Hrincius E Ludwig S Roth J Viemann D. Highly pathogenic avian influenza viruses inhibit effective immune responses of human blood-derived macrophages. J Leukoc Biol (2012) 92(1):11–20. 10.1189/jlb.0911479 22442495 PMC3382316

[85] van Riel D Leijten LM van der Eerden M Hoogsteden HC Boven LA Lambrecht BN Highly pathogenic avian influenza virus H5N1 infects alveolar macrophages without virus production or excessive TNF-alpha induction. PLoS Pathog (2011) 7(6):e1002099. 10.1371/journal.ppat.1002099 21731493 PMC3121882

[86] Chan MC Chan RW Chan LL Mok CK Hui KP Fong JH Tropism and innate host responses of a novel avian influenza A H7N9 virus: an analysis of ex-vivo and in-vitro cultures of the human respiratory tract. Lancet Respir Med (2013) 1(7):534–42. 10.1016/S2213-2600(13)70138-3 24461614 PMC7164816

[87] Davies LC Jenkins SJ Allen JE Taylor PR. Tissue-resident macrophages. Nat Immunol (2013) 14(10):986–95. 10.1038/ni.2705 24048120 PMC4045180

[88] Italiani P Boraschi D From monocytes to M1/M2 macrophages: phenotypical vs. functional differentiation. Front Immunol (2014) 5:514 10.3389/fimmu.2014.00514 25368618 PMC4201108

[89] Jegaskanda S Reading PC Kent SJ. Influenza-specific antibody-dependent cellular cytotoxicity: toward a universal influenza vaccine. J Immunol (2014) 193(2):469–75. 10.4049/jimmunol.1400432 24994909

[90] Moretta A Bottino C Vitale M Pende D Cantoni C Mingari MC Activating receptors and coreceptors involved in human natural killer cell-mediated cytolysis. Annu Rev Immunol (2001) 19:197–223. 10.1146/annurev.immunol.19.1.197 11244035

[91] Parham P Norman PJ Abi-Rached L Guethlein LA. Human-specific evolution of killer cell immunoglobulin-like receptor recognition of major histocompatibility complex class I molecules. Philos Trans R Soc Lond B Biol Sci (2012) 367(1590):800–11. 10.1098/rstb.2011.0266 22312047 PMC3267113

[92] Terajima M Co MD Cruz J Ennis FA. High antibody-dependent cellular cytotoxicity antibody titers to H5N1 and H7N9 avian influenza A viruses in healthy US adults and older children. J Infect Dis (2015) 212(7):1052–60. 10.1093/infdis/jiv181 25795791 PMC4668882

[93] Dusseaux M Martin E Serriari N Peguillet I Premel V Louis D Human MAIT cells are xenobiotic-resistant, tissue-targeted, CD161hi IL-17-secreting T cells. Blood (2011) 117(4):1250–9. 10.1182/blood-2010-08-303339 21084709

[94] Hazenberg MD Spits H. Human innate lymphoid cells. Blood (2014) 124(5):700–9. 10.1182/blood-2013-11-427781 24778151

[95] Tang XZ Jo J Tan AT Sandalova E Chia A Tan KC IL-7 licenses activation of human liver intrasinusoidal mucosal-associated invariant T cells. J Immunol (2013) 190(7):3142–52. 10.4049/jimmunol.1203218 23447689

[96] Chan AC Leeansyah E Cochrane A d’Udekem d’Acoz Y Mittag D Harrison LC Ex-vivo analysis of human natural killer T cells demonstrates heterogeneity between tissues and within established CD4(+) and CD4(-) subsets. Clin Exp Immunol (2013) 172(1):129–37. 10.1111/cei.12045 23480193 PMC3719939

[97] Paget C Ivanov S Fontaine J Renneson J Blanc F Pichavant M Interleukin-22 is produced by invariant natural killer T lymphocytes during influenza A virus infection: potential role in protection against lung epithelial damages. J Biol Chem (2012) 287(12):8816–29. 10.1074/jbc.M111.304758 22294696 PMC3308738

[98] Monticelli LA Sonnenberg GF Abt MC Alenghat T Ziegler CG Doering TA Innate lymphoid cells promote lung-tissue homeostasis after infection with influenza virus. Nat Immunol (2011) 12(11):1045–54. 10.1031/ni.2131 21946417 PMC3320042

[99] Qin G Mao H Zheng J Sia SF Liu Y Chan PL Phosphoantigen-expanded human gammadelta T cells display potent cytotoxicity against monocyte-derived macrophages infected with human and avian influenza viruses. J Infect Dis (2009) 200(6):858–65. 10.1086/605413 19656068 PMC7110194

[100] McMichael AJ Gotch FM Dongworth DW Clark A Potter CW. Declining T-cell immunity to influenza, 1977-82. Lancet (1983) 2(8353):762–4. 10.1016/S0140-6736(83)92297-3 6137602

[101] Sridhar S Begom S Bermingham A Hoschler K Adamson W Carman W Cellular immune correlates of protection against symptomatic pandemic influenza. Nat Med (2013) 19(10):1305–12. 10.1038/nm.3350 24056771

[102] Hayward AC Wang L Goonetilleke N Fragaszy EB Bermingham A Copas A Natural T cell-mediated protection against seasonal and pandemic influenza. Results of the Flu Watch Cohort Study. Am J Respir Crit Care Med (2015) 191(12):1422–31. 10.1164/rccm.201411-1988OC 25844934 PMC4476562

[103] Thomas PG Keating R Hulse-Post DJ Doherty PC. Cell-mediated protection in influenza infection. Emerg Infect Dis (2006) 12(1):48–54. 10.3201/eid1201.051237 16494717 PMC3291410

[104] Kedzierska K La Gruta NL Turner SJ Doherty PC. Establishment and recall of CD8+ T-cell memory in a model of localized transient infection. Immunol Rev (2006) 211:133–45. 10.1111/j.0105-2896.2006.00386.x 16824123

[105] Kedzierski L Linossi EM Kolesnik TB Day EB Bird NL Kile BT Suppressor of cytokine signaling 4 (SOCS4) protects against severe cytokine storm and enhances viral clearance during influenza infection. PLoS Pathog (2014) 10(5):e1004134. 10.1371/journal.ppat.1004134 24809749 PMC4014316

[106] Bird NL Olson MR Hurt AC Oshansky CM Oh DY Reading PC Oseltamivir prophylaxis reduces inflammation and facilitates establishment of cross-strain protective T cell memory to influenza viruses. PLoS One (2015) 10(6):e0129768. 10.1371/journal.pone.0129768 26086392 PMC4473273

[107] Kedzierska K Guillonneau C Gras S Hatton LA Webby R Purcell AW Complete modification of TCR specificity and repertoire selection does not perturb a CD8+ T cell immunodominance hierarchy. Proc Natl Acad Sci U S A (2008) 105(49):19408–13. 10.1073/pnas.0810274105 19047637 PMC2614774

[108] Hatta M Hatta Y Kim JH Watanabe S Shinya K Nguyen T Growth of H5N1 influenza A viruses in the upper respiratory tracts of mice. PLoS Pathog (2007) 3(10):1374–9. 10.1371/journal.ppat.0030133 17922570 PMC2000968

[109] Bao L Xu L Zhu H Deng W Chen T Lv Q Transmission of H7N9 influenza virus in mice by different infective routes. Virol J (2014) 11:185. 10.1186/1743-422X-11-185 25367670 PMC4289364

[110] Mok CK Lee HH Chan MC Sia SF Lestra M Nicholls JM Pathogenicity of the novel A/H7N9 influenza virus in mice. MBio (2013) 4(4):e00362-13. 10.1128/mBio.00362-13 23820393 PMC3705449

[111] Duan S Meliopoulos VA McClaren JL Guo XZ Sanders CJ Smallwood HS Diverse heterologous primary infections radically alter immunodominance hierarchies and clinical outcomes following H7N9 influenza challenge in mice. PLoS Pathog (2015) 11(2):e1004642. 10.1371/journal.ppat.1004642 25668410 PMC4335497

[112] Wilkinson TM Li CK Chui CS Huang AK Perkins M Liebner JC Preexisting influenza-specific CD4+ T cells correlate with disease protection against influenza challenge in humans. Nat Med (2012) 18(2):274–80. 10.1038/nm.2612 22286307

[113] Epstein SL. Prior H1N1 influenza infection and susceptibility of Cleveland Family Study participants during the H2N2 pandemic of 1957: an experiment of nature. J Infect Dis (2006) 193(1):49–53. 10.1086/498980 16323131

[114] Wright P Webster R Orthomyxoviruses. Philadelphia: Lippincott Williams & Wilkins (2001).

[115] Hillaire ML van Trierum SE Bodewes R van Baalen CA van Binnendijk RS Koopmans MP Characterization of the human CD8(+) T cell response following infection with 2009 pandemic influenza H1N1 virus. J Virol (2011) 85(22):12057–61. 10.1128/JVI.05204-11 21917970 PMC3209317

[116] Miao H Hollenbaugh JA Zand MS Holden-Wiltse J Mosmann TR Perelson AS Quantifying the early immune response and adaptive immune response kinetics in mice infected with influenza A virus. J Virol (2010) 84(13):6687–98. 10.1128/JVI.00266-10 20410284 PMC2903284

[117] Ennis FA Rook AH Qi YH Schild GC Riley D Pratt R HLA restricted virus-specific cytotoxic T-lymphocyte responses to live and inactivated influenza vaccines. Lancet (1981) 2(8252):887–91. 10.1016/S0140-6736(81)91389-1 6117682

[118] Wagar LE Rosella L Crowcroft N Lowcock B Drohomyrecky PC Foisy J Humoral and cell-mediated immunity to pandemic H1N1 influenza in a Canadian cohort one year post-pandemic: implications for vaccination. PLoS One (2011) 6(11):e28063. 10.1371/journal.pone.0028063 22132212 PMC3223223

[119] Jameson J Cruz J Terajima M Ennis FA. Human CD8+ and CD4+ T lymphocyte memory to influenza A viruses of swine and avian species. J Immunol (1999) 162(12):7578–83. 10358215

[120] Goy K Von Bibra S Lewis J Laurie K Barr I Anderson D Heterosubtypic T-cell responses against avian influenza H5 haemagglutinin are frequently detected in individuals vaccinated against or previously infected with human subtypes of influenza. Influenza Other Respir Viruses (2008) 2(4):115–25. 10.1111/j.1750-2659.2008.00046.x 19453462 PMC4634225

[121] Kreijtz JH de Mutsert G van Baalen CA Fouchier RA Osterhaus AD Rimmelzwaan GF. Cross-recognition of avian H5N1 influenza virus by human cytotoxic T-lymphocyte populations directed to human influenza A virus. J Virol (2008) 82(11):5161–6. 10.1128/JVI.02694-07 18353950 PMC2395172

[122] Lee LY Ha do LA Simmons C de Jong MD Chau NV Schumacher R Memory T cells established by seasonal human influenza A infection cross-react with avian influenza A (H5N1) in healthy individuals. J Clin Invest (2008) 118(10):3478–90. 10.1172/JCI32460 18802496 PMC2542885

[123] van de Sandt CE Kreijtz JH de Mutsert G Geelhoed-Mieras MM Hillaire ML Vogelzang-van Trierum SE Human cytotoxic T lymphocytes directed to seasonal influenza A viruses cross-react with the newly emerging H7N9 virus. J Virol (2014) 88(3):1684–93. 10.1128/JVI.02843-13 24257602 PMC3911609

[124] Quinones-Parra S Grant E Loh L Nguyen TH Campbell KA Tong SY Preexisting CD8+ T-cell immunity to the H7N9 influenza A virus varies across ethnicities. Proc Natl Acad Sci U S A (2014) 111(3):1049–54. 10.1073/pnas.1322229111 24395804 PMC3903243

[125] Kedzierska K Turner SJ Doherty PC. Conserved T cell receptor usage in primary and recall responses to an immunodominant influenza virus nucleoprotein epitope. Proc Natl Acad Sci U S A (2004) 101(14):4942–7. 10.1073/pnas.0401279101 15037737 PMC387353

[126] Marshall DR Turner SJ Belz GT Wingo S Andreansky S Sangster MY Measuring the diaspora for virus-specific CD8+ T cells. Proc Natl Acad Sci U S A (2001) 98(11):6313–8. 10.1073/pnas.101132698 11344265 PMC33465

